# Emerging roles of chicken and viral microRNAs in avian disease

**DOI:** 10.1186/1753-6561-5-S4-S2

**Published:** 2011-06-03

**Authors:** Joan Burnside, Robin Morgan

**Affiliations:** 1 Delaware Biotechnology Institute, University of Delaware, 15 Innovation Way, Newark, Delaware 19711, USA

## Abstract

**Abstract:**

**Background**

MicroRNAs are short RNAs (~22 nt) expressed by plants, animals and viruses that regulate gene expression post-transcriptionally, and their importance is highlighted by distinct patterns of expression in many physiological processes, including development, hematopoeisis, stress resistance, and disease. Our group has characterized the microRNAs encoded by the avian herpesviruses; namely, oncogenic Marek’s disease (MD) virus (MDV1), non-oncogenic MDV (MDV2) herpesvirus of turkeys (HVT), and infectious laryngotracheitis virus (ILTV).

**Methods**

MicroRNAs encoded by the avian herpesviruses were identified using next generation sequencing technologies (454, Illumina).

**Results**

The microRNAs of each the avian herpesviruses have unique sequences, but the genomic locations are similar, in that the microRNAs tend to be clustered in the rapidly evolving repeat regions of the viral genomes. For a given viral species the microRNA sequence is highly conserved in different strains with the exception of a virulence-associated polymorphism in the putative promoter of the MDV1 microRNAs upstream of the *meq* oncogene. These microRNAs are relatively highly expressed in tumors produced by very virulent MDV1 isolates compared to tumors produced by less virulent strains. MDV1 and HVT encode homologs of the host microRNA, miR-221, which targets a gene important in cell cycle regulation. MDV1 encodes a microRNA (mdv1-miR-M4) that shares a seed sequence with miR-155, a microRNA important in immune function. Mdv-miR-M4 is highly expressed in MDV induced tumors, while miR-155 is present at very low levels.

**Conclusions**

MicroRNAs are highly conserved among different field strains of MDV1, and they are expressed in lytic and latent infections and in MDV1-derived tumors. This suggests that these small molecules are very important to the virus, and roles in immune evasion, anti-apoptosis, or proliferation are likely.

## Background

Marek's disease (MD) is a lymphoproliferative disorder in which aggressive T-cell lymphomas develop within two to six weeks following infection of susceptible chickens with oncogenic MD virus (MDV1) [1]. The molecular details of MDV1-induced transformation are not well understood, but vaccines developed from related, nononcogenic strains, such as MDV2 and herpesvirus of turkeys (HVT), protect animals from disease. Thus, MD is unique among virally induced lymphomas in that highly effective vaccines exist. Despite vaccination, MD continues to be a problem worldwide, and new viruses with enhanced virulence have emerged over the last three decades. This is explained, in part, because vaccines prevent disease but not infection, and vaccinated chickens shed virulent virus into the environment. MDV1 is immunosuppressive, and one explanation for enhanced virulence of recently emerged strains is that the virus has evolved to become increasingly competent at immune system disarmament or evasion.

MDV1 is an α-herpesvirus, and infection includes both lytic and latent stages. Productive infection is characterized by the replication of viral DNA, the synthesis of viral proteins, and ultimately the assembly of infectious virus. In the natural host, fully enveloped, infectious virus is produced only from feather follicle epithelium. A restrictive productive infection occurs in B cells and chicken embryo fibroblasts (CEF) in culture, and is characterized by viral replication and viral antigen synthesis but no fully infectious virus particle production. MDV1 assumes a latent posture in T lymphocytes, with little expression of viral antigens. T lymphocytes can be transformed by MDV1, and the virus in transformed cells is generally considered to be latent, but several viral genes are transcribed in tumors as well as in lymphoblastoid cell lines derived from them. This includes *meq*, the candidate oncogene for MDV1 [2], as well as the latency associated transcripts (LATs) [3], which map antisense to the major transcriptional activator ICP4 and do not encode a protein product. MDV2 is a related, non-oncogenic virus that infects chickens. HVT is apathogenic and primarily infects turkeys, but has been used in combination with MDV2 as a very effective vaccine against MDV1. ILTV is more distantly related to MDV and HVT, and causes a respiratory illness in chickens.

*microRNAs.* MicroRNAs are short RNAs (~22 nt) that are important post-transcriptional regulators encoded by the genomes of animals and plants [4]. Over 940 microRNAs have been discovered in the human genome [5], and genes encoding microRNAs have distinct expression profiles during development, in specific tissues, in disease, and in response to stimuli (*for a recent review*, *see*[*6*]). MicroRNAs are transcribed by RNA polymerase II into long primary microRNA (pri-microRNA) transcripts, which are capped and polyadenylated and can fold into characteristic hairpin structures. The pri-microRNA, which can encode either a single microRNA or a cluster of several, is processed by a ribonuclease III-like enzyme, Drosha, resulting in the liberation of a 60- to 70-nucleotide RNA hairpin (pre-microRNA). The pre-microRNA is exported to the cytoplasm and further processed into a short dsRNA molecule by a second ribonuclease III-like enzyme, Dicer. A single strand of this short dsRNA, the mature microRNA, is incorporated into the RNA-induced silencing complex (RISC), which includes the endonuclease Argonaute, while the other strand, known as the star or passenger strand, is usually degraded. The RISC complex directs transcript cleavage or translational repression of target genes, and it is generally thought that the choice of mechanism depends on the degree of complementarity between the microRNA and its target site on an mRNA. In plants, microRNAs show high complementarity to their target mRNAs, and this results in endonucleolytic transcript cleavage known as ‘slicing’ [7]. In animals, complementarity between nucleotides 2-7/8 of the microRNA (known as the ‘seed’ sequence) and the 3’UTR of the target mRNA appears to be crucial for directing translational repression, the predominant mechanism for microRNA action in animals (*reviewed in*[*8*]).

Herpesviruses also encode microRNAs. Herpesviruses have developed strategies to evade the host immune response during both lytic replication and persistent latent infections. Viral protein coding genes carry out varied functions important to viral survival such as blocking interferon responses, inhibiting apoptosis and interfering with innate and adaptive immune responses. The microRNAs encoded by herpesviruses are now receiving attention as critical regulators of both viral and host genes and are like to play key roles in viral survival [9].

We postulated that MDV1 and other avian herpesviruses (MDV2, HVT and infectious laryngotracheitis virus (ILTV) encode microRNAs; this paper reviews current information about avian herpesvirus microRNAs and their roles.

## Methods

### Deep sequencing and sequence analysis

Total RNA was purified using Trizol (Invitrogen, Carlsbad, CA), and small RNAs were purified, and cloned, and submitted to 454 (Branford, CT) or to Illumina (Hayward, CA) for sequencing. Sequence reads were compared to the chicken and viral genomes, allowing only exact matches using an in-house developed analysis pipeline. After filtering out other known small RNAs, candidate microRNA sequences mapping to unique loci were tallied to determine sequencing read frequency.

### Cell cultures and viruses

CEF were prepared as described previously [10,11]. MDV1, MDV2 and HVT strains were from the University of Delaware collection, ILTV USDA was from ATCC via the University of Delaware collection.

### RNA preparation and northern blotting

RNA was purified using Trizol, electrophoresed on acrylamide gels, and hybridized to ^32^P-labeled antisense oligonucleotide probes as described previously [12].

## Results and discussion

### MDV1, MDV2, HVT, and ILTV encode microRNAs

**Figure 1:**
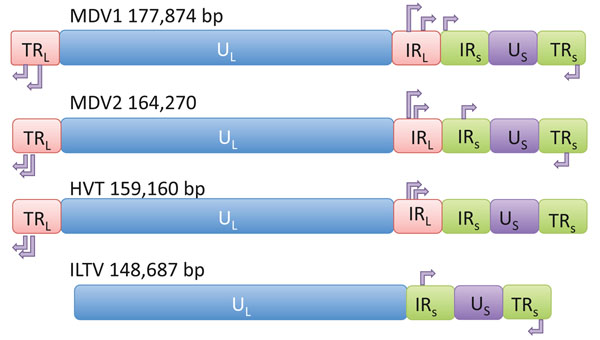
**Schematic representation of avian herpesviruses and location of clusters of microRNAs.** Terminal and internal short and long repeats are designated TRS, IRS, TRL and TRS. The approximate location and direction of transcription of microRNA clusters are indicated by the arrows. Lengths of each region are not drawn to scale.

Our laboratory has identified microRNAs encoded by four avian herpesviruses, MDV1, MDV2, HVT, and ILTV [13,14]. Like other herpesviruses, the genome of the avian herpesviruses contains unique long (U_L_) and unique short (U_S_) regions that are flanked by terminal and internal repeats (I/TR_L_; I/TR_S_). The unique regions contain functionally conserved protein coding sequences, while the repeats generally encode virus-specific genes. The avian herpesvirus microRNAs tend to be clustered in these highly plastic, repeat regions (Figure 1). In accordance with these being virus-specific regions, there is no conservation of sequence among any of the microRNAs from the different viruses. We have speculated that the viral microRNAs appeared with the evolution of the repeat regions, suggesting that the function of the microRNAs is to provide an advantage to the virus, and if their acquisition produced a loss-of-function, this could be complemented by sequences in the other repeat. Gain-of-function mutations, however, would be maintained and eventually duplicated.

In MDV1, microRNAs either flank the *meq* oncogene or lie in the region encoding the LATs [13,15]. The predominant ILTV microRNAs are antisense to the ICP4 gene, which encodes an essential immediate early transcriptional activator, and so ILTV microRNAs could serve to regulate ICP4 mRNA levels [16]. It is interesting that several of the HVT microRNAs appear to have arisen from local duplications of portions of the viral genome [14]. A striking sequence similarity between two MDV1 microRNAs (mdv1-miR-M7, M10) suggests that they also may have been generated by local duplication within the genome.

### Viral microRNAs are highly conserved among strains

In contrast to the lack of sequence conservation among the different avian herpesviruses, the microRNAs of the different viral strains are highly conserved.

In MDV1, the sequence of all microRNAs was conserved among 23 different strains representing three pathotypes. However, one polymorphism in the putative promoter for the *meq* microRNA cluster correlates with pathogenicity, and could be responsible for the difference in expression of the *meq* microRNAs in tumors from very virulent pathotypes [17] (Table 1).

**Table 1:** Relative expression levels of MDV1 microRNAs in tumors produced by a very virulent + strain (vv+, 615K) compared to a very virulent strain (vv, RB1B) strain

microRNA	**vv**^+^**/vv**
**Upstream of meq**	
mdv1-miR-M2-5p	3.9
mdv1-miR-M2-3p	6.4
mdv1-miR-M3-5p	1.6
mdv1-miR-M4-5p	3.3
mdv1-miR-M5-3p	3.2
mdv1-miR-M12-3p	2.3

**LAT region**	

mdv1-miR-M6-5p	0.9
mdv1-miR-M6-3p	1
mdv1-miR-M7-5p	1.1
mdv1-miR-M7-3p	0.7
mdv1-miR-M8-5p	0.9
mdv1-miR-M8-3p	0.9

Ratios were derived from average densitometry of northern blot analysis of samples from two different chickens for each strain. Expression of viral DNA was comparable in all samples. U6 was used as a loading control and showed a ratio of 0.8.

Only two strains of HVT (FC126, PB1) and two MDV2 strains (SB1 and HPRS-24) are available for sequence comparison. In preliminary studies, no polymorphisms have been identified in microRNAs of MDV2, while three polymorphisms were found in HVT PB1 microRNAs (hvt-miR-H3-5p;12-5p;16-5p) compared to the reference strain FC126. In both hvt-miR-H12-5p and 16-5p, the polymorphism is located within the seed sequence. Similarly, the sequence of the predominant ILTV microRNAs, iltv-miR-I5 and I6, are highly conserved; these microRNAs lie antisense to the ICP4 gene and this region is identical in the 22 strains of ILTV included in GenBank.

Overall, the high level of conservation supports the idea that there is selective pressure to maintain the microRNA sequences, inferring that viral microRNAs have important functions [18]. Despite the lack of sequence identity among the microRNAs of the different avian herpesviruses, there may be overlaps in function. Typically, a microRNA binds to the 3’UTR of target genes, with binding mediated through the ‘seed’ sequence (nt 2-7/8) of the microRNA [19]. Thus, the same gene could be targeted at different sequences by different microRNAs, and some of these divergent avian herpesviral microRNAs could share targets.

### Viral orthologs of host microRNAs

One of the HVT microRNAs, hvt-miR-H14-3p, is a homologue of gga-miR-221 with 21/23 nucleotide identity, including complete conservation of the seed/family sequence. There is sequence similarity with downstream flanking region in the chicken genome as well [14]. In the chicken, miR-221 is very abundant in CEFs and other tissues [12,20,21], and it is likely that expression is similar in the turkey, which is the natural host of HVT. Thus it is possible that this HVT homologue was captured from the host genome, either through propagation in tissue culture or during infection and replication in the host. MDV1 contains a microRNA (mdv1-miR-M32) that shares a seed sequence with miR-221 {Morgan, 2008 #4818}. miR-221 targets the cyclin-dependent kinase inhibitor 1B (p27, Kip1), a regulator of the cell cycle G1 to S phase transition. The repression of p27 by miR-221 is thought to play an important role in cancer progression [22,23], and could play a role in MDV1-induced tumorigenesis {Lambeth, 2009 #5006}. Although HVT is non-oncogenic, expression of a miR-221 ortholog and down-regulation of p27 could move the cell cycle to the S phase in order to support replication of the viral genome as well as to increase growth of infected cells for additional viral production.

MDV1, like Kaposi’s sarcoma herpesvirus (KSHV), encodes a microRNA that shares a seed sequence with the host microRNA, miR-155 (kshv-miR-K11; mdv1-miR-M4). It has been proposed that kshv-miR-K11 is a functional ortholog of mir-155 and can regulate the same set of host mRNAs, and this contributes to KSHV-induced tumorigenesis [24], [25]. Because the host response to infection must be balanced between elimination of pathogens and limited damage to host cells, microRNAs are ideal candidates for fine-tuning of the rapidly changing immune response. However, the timing and cellular location of expression are critical for manifesting function of immunomodulatory microRNAs. For miR-155, loss- and gain-of-function studies have demonstrated a critical role in both immune cell development and function. Sustained expression of miR-155 can stimulate production of granulocytes, and miR-155 over-expression can lead to neoplasia [26]. However, miR-155 expression is complicated in that its induction in macrophages by activation of the immune response with lipopolysaccharides does not result in subsequent neoplasia [27,28]. Mdv1-miR-M4 also shares some targets with mir-155, and it has been suggested that mdv1-miR-M4 target regulation plays a role in MDV-induced lymphomagenesis [29]. In deep sequencing studies of MDV-induced splenic tumors, we have noted decreased levels of miR-155 (and most microRNAs) compared to normal spleen, resting T cells, or activated T cells (Table 2). An overall decline in microRNA expression has been noted in other cancers [30,31], and we hypothesize that mdv1-miR-M4 serves to replace miR-155 in regulating functions essential to the phenotype of the transformed cell. In addition, as noted above, tumors produced by more aggressive strains of MDV1 express higher levels of mdv1-miR-M4 and other microRNAs in this cluster (Table 1), consistent with a role in tumorigenesis. To further elucidate the role of mdv1-miR-M4, our laboratory has constructed arecombinant HVT that expresses mdv-miR-M4. The recombinant virus showed improved growth characteristics *in vitro* and *in vivo* implying that this microRNA can confer replicative advantages to the virus, but mdv1-miR-M4 is not turmorigenic in the context of HVT (unpublished results). Thus the MDV1 ortholog of miR-155 may also be pleomorphic in that it facilitates viral growth by impeding the apoptotic response to infection and also down-regulates genes involved in cell cycle control.

**Table 2:** Sequencing frequencies of immune-related (A) and MDV (B) microRNAs in spleen, T cells, activated T cells (a-T), and MDV-induced splenic tumors

	spleen	T cells	a- T cells	tumor
**A**				
gga-miR-21	15779	4441	3002	693
gga-miR-181a	2626	1507	11602	54
gga-miR-181b	727	956	9458	59
gga-miR-221	2326	2679	13295	83
gga-miR-222	324	1166	21646	72
gga-miR-146a	794	407	1165	36
gga-miR-146b	2154	1016	11865	857
gga-miR-155	499	1403	52081	29
gga-miR-92	973	1943	1799	211

**B**				
mdv-miR-M2-5p				104
mdv-miR-M2-3p				591
mdv-miR-M3-5p				187
mdv-miR-M3-3p				50
mdv-miR-M4				6682
mdv-miR-M5				581
mdv-miR-M8-5p				345
mdv-miR-M9-5p				63
mdv-miR-M9-3p				74
mdv-miR-M12-3p				164
mdv-miR-M10-5p				12

## Conclusions

The presence of microRNAs in species-specific repeat regions of the genomes of avian herpesvirus and the conservation among strains in a particular virus all point to the importance of these gene regulators in virus survival. While the functions of the viral microRNAs have not been established, it is apparent that these are ideally designed for fine-tuning the host response to infection with viruses that replicate and also form persistent latent infections in the host. Maintaining cell viability and establishing a permissive environment for unregulated synthesis of foreign DNA requires controlling the cell cycle, blocking apoptosis and escaping the innate immune response to infection; genes regulating these pathways are among the potential targets for these molecules. In addition, microRNAs are emerging as therpeutic targets, and understanding their roles in viral infection has great potential for the development of improved treatments and vaccines.

## Competing interests

The authors declare that they have no competing interests.

## Authors' contributions

JB and RM directed the research and wrote the manuscript.
